# Movement of bacteria in the soil and the rhizosphere

**DOI:** 10.1128/aem.00246-25

**Published:** 2025-09-12

**Authors:** Gladys Alexandre

**Affiliations:** 1 Department of Biochemistry and Cellular and Molecular Biology, University of Tennessee 4292, Knoxville, Tennessee, USA; University of Delaware, Lewes, Delaware, USA

**Keywords:** fungal highways, bacterivorous predators, hitchhiking, rhizosphere, soil, swimming, social spreading

## Abstract

The soil and the rhizosphere are physicochemically heterogeneous environments that host diverse macro- and micro-organisms that together influence soil productivity. The ability of organisms to disperse in these environments allows them to exploit resources and to occupy niches that support growth and protect them from predation and stressful conditions. The dispersal of soil macroorganisms has been much better characterized than that of bacteria because of the complexity and physicochemical heterogeneity of the soil and the rhizosphere, and challenges in quantifying the dispersion of bacteria in these environments. However, even limited bacterial dispersal in soils and the rhizosphere could have the potential to alter the local microbiome composition and its function. Active bacterial movement includes swimming and swarming using flagella, twitching motility using pili, as well as emerging forms of motility that result from microbe-microbe interactions. Passive transport of bacteria throughout the soil may be mediated by passive physical factors such as rainfalls, as well as through transport mediated by protists, nematodes, or hitchhiking using other microbes’ appendages. This minireview focuses on the modes of bacterial movement in the soil and the rhizosphere that do not depend on passive physical factors (e.g., rainfalls) and identifies areas of future research.

## INTRODUCTION

The soil and the rhizosphere (defined as the narrow region of soil around the roots that is directly influenced by root secretions per Hiltner’s definition [1]) host thousands of different species that are critical to nutrient cycling and plant productivity, as well as to Earth’s biogeochemical cycles (2). The soil microbial biomass consists of bacteria, archaea, fungi, algae, and protozoa (3). The soil environment is physically and chemically heterogeneous, with the soil type and texture influencing pore and aggregate sizes, density, and structures contributing to producing a range of microhabitats for soil microorganisms (4). Soil microorganisms and their interaction with each other and other organisms (e.g., plant roots) contribute to modifying soil chemistry to further shape the soil microhabitats (2). The presence of plants modifies the surrounding environment through root exudation and modulation of the local microbial activities and contributes to structuring the rhizosphere soil (5). The physicochemical soil heterogeneity is further modulated by the soil water content. For example, after heavy rains, water saturation does not necessarily translate into soil pores being filled with water. This is because pore geometry and soil mineralogical composition can affect hydraulic conductivity as a result of the combined effects on capillary forces exerted at the pore surfaces and permeability through the pores (6). Therefore, soils always comprise significant pore volumes that are air-filled. Soil microorganisms live in a thin layer of water close to pore surfaces, with the size of that layer depending on water availability and physicochemical properties of the pores that can modulate hydrophobicity of the surfaces (2, 6). The activity of soil microbes depends on water availability, and the fraction of active microbes will then depend on the complex interactions between soil physicochemical properties, water content, and the presence of plants. In the rhizosphere, microbial activity and density are elevated and supported by root secretions, including mucilage and root exudates (5). The chemical composition of the rhizosphere is also heterogeneous due to the existence of spatiotemporal gradients originating from the roots, as well as contributed by the activity of rhizosphere microbes that can change quickly with water conditions or shifts in water availability and plant developmental stages. The dispersion of bacteria in the soil and the rhizosphere provides soil ecosystem services since it affects nutrient cycling, microbial communities’ activity in the soil and the rhizosphere (5). Recent studies of the soil microbes’ biogeography analyzed through high-throughput 16S rDNA sequencing indicate that bacterial dispersal in soils is likely limited (7, 8). However, even limited bacterial dispersal in soils and the rhizosphere could have the potential to alter the local microbiome composition and its function (9–12). However, studying the dispersion of bacteria directly in the soil or in the rhizosphere remains difficult largely because of the complexity and physicochemical heterogeneity of the soil and the rhizosphere, and challenges in quantifying dispersion in these environments.

Few studies have directly addressed the dispersal of bacteria in soils, and in these few cases, the dispersion of bacteria was analyzed as a function of water saturation (13–16). The ability of bacteria to disperse in the soil depends on both active and passive mechanisms. Active bacterial movement includes swimming and swarming using flagella, with direction of movement guided by chemotaxis signaling (17, 18). Twitching motility, which describes a movement dependent on the extension and retraction of polar type IV pili, modulates the interaction of bacteria with host plant roots (19–22) and plays a role in the formation of biofilms on plant root surfaces or within plant tissues (23, 24). In this respect, the role of twitching motility in transport within the soil or the rhizosphere may be limited. Emerging forms of motility that result from microbe-microbe interactions have also been recently described. Passive transport of bacteria throughout the soil may be mediated by passive physical factors such as rainfalls, which are likely a major mode of bacterial dispersal through the soil horizons (25–27). Bacteria can also disperse on soil surfaces through air, leaf litter, and through the introduction of new vegetation (28). Dispersal through leaf litter appears to contribute significantly to the dispersal of bacteria in some environments (28). Passive mechanisms of bacterial dispersion in the soil and the rhizosphere also include transport mediated by protists, nematodes, as well as hitchhiking using other microbes’ appendages. While the mechanisms of movement of diverse soil and rhizosphere bacteria have been studied in the laboratory, little information is available regarding the impact and contribution of different modes of movement (passive or active) on the dispersion and distribution of bacteria in the soil and the rhizosphere. Biological movements in the soil span a range of speeds depending on the organism considered and the movement mechanism (Table 1). Instead of focusing on the ecology of microbial dispersal, this minireview focuses on the mechanisms of movement and their potential for contributing to bacterial dispersal. This minireview discusses modes of bacterial movement in the soil and the rhizosphere that do not depend on passive physical factors (e.g., air, rainfalls) and finally, highlights areas where additional research is needed to integrate with our understanding of soil microbial community functions.

**TABLE 1:** Speed rates of biological movements occurring in the soil and discussed in the minireview

Type of movement	Movement speed range (μm/s)	Reference
Swimming bacteria	20–100	(29)
Swarming, gliding, twitching bacteria	0.1–10	(29)
Protist motility	0.1–180	(30)
Hyphal extension rates (root fungal pathogens)	0.2–50	(31)
Plant root growth rates	0.09–0.8	(31)

## DISPERSION OF MOTILE BACTERIA USING FLAGELLA

**Fig 1:**
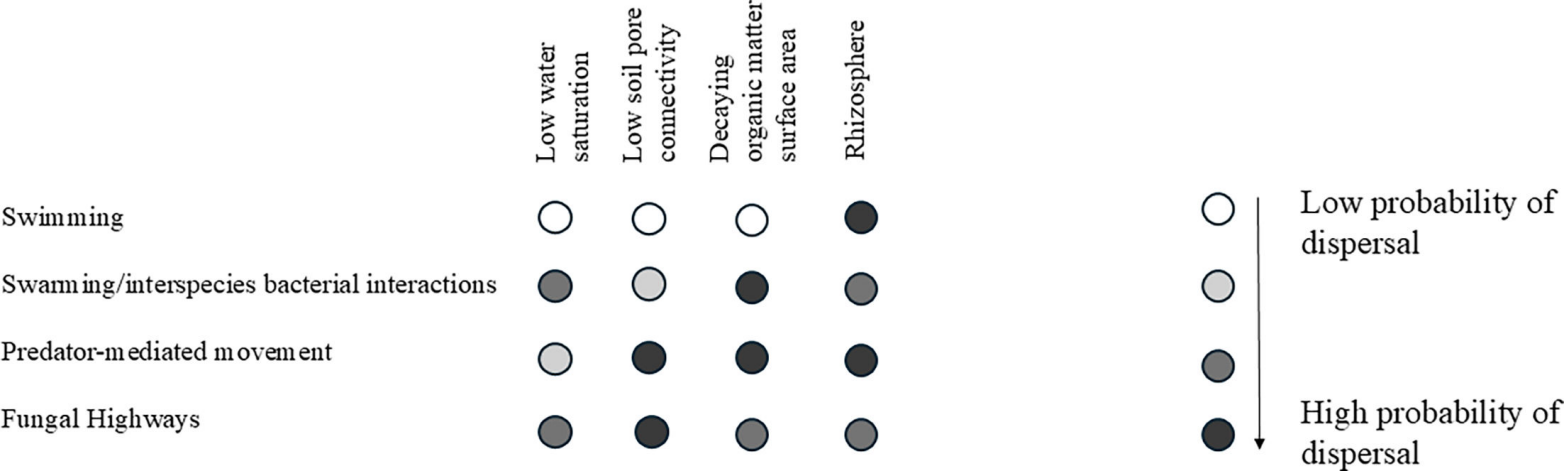
Summary of the potential for bacterial dispersal in the soil and the rhizosphere as a function of the edaphic conditions and the mode of transport. The modes of transport are described in the text. Predator-mediated movement includes movement caused by nematodes and protists as bacterial predators. Swimming and swarming refer to bacterial flagella-mediated motility in liquid or viscous conditions, respectively.

Bacterial motility by flagella has been well studied in various soil and plant-associated bacteria (17). The direction of movement of flagellated motile bacteria is controlled by a (or multiple) conserved chemotaxis signaling system that links sensing of environmental cues to changes in the direction of swimming to navigate gradients of chemoeffectors (17). Flagellar motility is likely advantageous in the soil and the rhizosphere because this trait is enriched in these environments (17, 32–34). Flagellar motility and chemotaxis responses allow motile cells to quickly navigate toward nutrient sources such as root surfaces or decaying organic matter (17, 34) (Table 1). Bacterial flagellar motility is more prevalent in soils with higher carbon availability and in the rhizosphere, which is also rich in available rapidly metabolizable sources of carbon (34). Given the heterogeneity of water distribution in the soil and the uneven connectivity between soil pores, especially under limiting water conditions, the movement of flagellated bacteria may not contribute to dispersion over large distances in soils unless water saturation is sufficient to promote this dispersion (Fig. 1). Conditions are likely different in the rhizosphere because of the presence of mucilage that retains water around the roots, as well as the active absorption of water, especially through root hairs. These conditions can produce a thin film of water in close proximity to the root surfaces that could support bacterial flagellar motility along the root surfaces. By influencing water flow, the density of root hairs could also then influence water available for bacterial swimming and thus the extent of bacterial dispersion within the rhizosphere or along the roots. Experimental evidence that the presence of flagellar motility provides competitive colonization in the rhizosphere fully supports that flagellar motility contributes to the movement of bacteria in this environment (17, 32, 34). Most studies using model systems and mutant strains have shown that swimming motility and chemotaxis play critical roles for the competitiveness of soil bacteria in the rhizosphere and in the establishment of mutualistic, neutral, or pathogenic interactions between bacteria and diverse host plant roots (17, 35–38). The type of bacterial movement in fluids depends on the fluid’s viscosity, i.e., its resistance to flow. Liquid water has low viscosity and enables bacterial swimming motility. Swimming motility occurs under conditions of low viscosity (e.g., liquid media under laboratory conditions). Swimming motility thus relies on the presence of a continuous film of water to allow dispersion over long distances. In contrast, swarming motility occurs under increased viscosity on surfaces (or on the surfaces of semi-soft agar plates under laboratory conditions) and propels groups of cells that produce many peritrichous flagella (18). Many soil bacteria are flagellated and likely capable of swarming (18, 34). However, the contribution of swarming to the dispersion of bacteria in the soil and the rhizosphere is not clear. Mucilage or any high polymer content molecules, such as polysaccharides (e.g., extracellular polysaccharides [EPS]), increased the viscosity of the soil water phase (39, 40). It is thus likely that in the rhizosphere, where plant mucilage and other microbially-derived polymeric substances are abundant, they could increase soil or rhizosphere liquid phase viscosity (41) and possibly induce swarming of soil bacteria. *Paenabacillus vortex* is found in the soil and the rhizosphere and is a robust swarmer that can move under high viscosity conditions (42). *P. vortex* can produce spores that it can transport on its own flagella as a bet-hedging strategy. *P. vortex* can also transport fungal conidia of *Aspergillus fumigatus* (43). The latter transport can move spores over long distances (tens of centimeters) (43). The association between *P. vortex* and *A. fumigatus* appears relatively specific. The transport of *A. fumigatus* conidia by the *P. vortex* swarms is mutually beneficial: the transport facilitates fungal conidia dispersal, can rescue conidia from adverse conditions, while the mycelia from the fungus could assist the bacteria in crossing otherwise impenetrable air-filled gaps in soil pores (43).

## PASSIVE MOVEMENTS OF BACTERIA ARE DEPENDENT ON OTHER ORGANISMS’ BEHAVIORS

Hitchhiking, or the process by which swarming bacterial transport spores or non-motile organisms, is a form of passive movement since the association between the flagella and the surfaces of the spores or cells transported is apparently not a specific process. Various species of non-motile Streptomycetes can be transported on the flagella of swarming *Bacillus subtilis* and *Pseudomonas fluorescens* (44). While the dispersal of Streptomycetes’ spores on the *B. subtilis* flagella depends in part on the conserved spore coat rodlin proteins, these are not required for the transport, suggesting the association between Streptomycetes’ spores and bacterial flagella is not specific (44). This mode of dispersal allowed *Streptomyces coelicolor* to colonize the roots of *Arabidopsis thaliana*, highlighting a potential ecological relevance (44). The lack of specificity between Streptomycetes and bacterial flagella is likely beneficial for efficient dispersal under conditions that promote bacterial swarming. Similarly, *P. vortex* can transport other cells unable to move across high viscosity surfaces, such as bacterial cells of *Xanthomonas perforans,* which are non-motile (45).

Soil-dwelling protists and nematodes are bacterial and fungal predators that play critical roles in litter decomposition, carbon and nutrient cycling, as well as in plant health (46–49). While the diverse soil and rhizosphere bacterial and fungal communities have been well-characterized, challenges in characterizing the diverse communities of soil-dwelling protists and nematodes remain (50, 51). The challenges result from the current limited genomic and molecular resources to detect these organisms *in situ,* such as the lack of universal primers and the small number of complete genome sequences available for these organisms, though recent improvements in these methods have been reported (52, 53). Protists and nematodes preying on bacteria, archaea, and fungi have species-specific feeding preferences that can change microbiome composition and functioning (54–56). Microbes have evolved various strategies to avoid predation, including the production of secondary metabolites and extracellular enzymes toxic to the eukaryotic (protists or nematodes) predators (57); morphological adaptation, such as changes in cell surface properties and formation of microcolonies (against protists); biofilm formation (against nematodes) (58–60); or filamentation or aggregations of cells into large clusters (against protists) (59, 60). In addition, motile bacteria are able to avoid bacterivorous protists by increasing their cell swimming speed (30). The latter avoidance swimming response of motile bacteria away from protist predators could explain the enhanced transport of *Sinorhizobium meliloti* along the roots of *Medicago truncatula,* recently reported, and allowing the formation of root nodules deeper in the soil horizon (61, 62).

Bacterivorous protists and nematodes also transport and promote the dispersal of bacteria in the soil (Fig. 1). Bacteriovorus nematodes can transport living bacteria within their intestines and release them through defecation at distances from where these microorganisms were ingested (63, 64). Diet preferences of bacterivorous nematodes, which preferentially prey on Gram-negative and non-pathogenic bacteria (65, 66), could influence the bacteria transported. Furthermore, bacteria can be moved by the feeding currents of protists, especially ciliates, while others can be transported as “backpacks” on the surface of protists (67–69). Predation-resistant bacteria are either resistant to phagocytosis and will be egested or can survive within the phagosome, either transiently or, for some bacterial genera, more permanently as endosymbionts (70–73). Unedible and predation-resistant bacteria could also be transported as cargo within protists. For example, *Dictyostelium discoideum* stably associates with endosymbionts of the genus *Burkholderia* through a symbiotic association defined as “proto-farming” (71, 72). Proto-farming may also mediate the transport of the endosymbiont throughout the soil or in the rhizosphere. Diverse bacteria can also attach to the cuticles of both bacteriovorus and non-bacteriovorus nematodes and become dispersed as the nematodes move through the soil (74). The cysts of *Heterodera* and *Globodera* nematodes harbor diverse microbiomes and could disperse the associated microbiota upon cyst germination (75, 76). Under optimum edaphic conditions and with sufficient water saturation, both nematodes and protists could travel long distances (several centimeters) through the soil. However, the impact of nematodes and protist-mediated transport of bacteria through the soil and the rhizosphere remains largely unexplored. Predatory bacteria also use type IV pili-mediated twitching motility to prey on target organisms on surfaces (77, 78). Predation could thus promote the movement of the predator on the prey-containing surfaces and possibly trigger the prey to disperse away from the predator, though this movement is likely limited unless there is sufficient water saturation to allow the fleeing of the motile prey to disperse further away from the biofilm (Fig. 1).

## DISPERSAL THROUGH FUNGAL HIGHWAYS

In addition to swimming, bacterial flagella-mediated motility allows the dispersion of soil bacteria on fungal mycelia (79–81). The dispersal of (motile and non-motile) bacteria through the fungal hyphae networks was coined as fungal highways (82). This mode of transport may be particularly significant in the soil and in linking the roots of various plants. The mycelia of mycorrhizal fungi form extensive networks (up to 20,000 km in 1 cubic meter of soil) that release soil phosphorus and nitrogen, as well as other nutrients that can then be absorbed by soil organisms and plants (83). Movement of bacteria within fungal highways can disperse bacteria over air-filled soil pores or bridge the roots of distant plants (80–82). Dispersion of bacteria on fungal highways appears to be widespread because diverse fungi and bacteria can be identified within such interactions (84). Bacterial flagellar motility on fungal highways occurs within a thin film of water present around the fungal hyphae, and both swimming and swarming bacteria appear to be able to disperse on fungal highways (84). Bacterial movement along the fungal hyphae may be in the same direction as the hyphal growth axis or opposite to it (84). The speed of a population of swimming *Pseudomonas putida* cells along the hyphae of *Pythium ultimum* was recently evaluated to include both slow-moving (~2 µm/s) and fast-moving cells (22–25 µm/s) (85). Some evidence suggests that fungal mycelia preferentially recruit some bacteria to their hyphae: the ability of bacteria to utilize particular compounds found in fungal exudates and/or to be chemotactically attracted to fungal exudates appears to promote and enhance their dispersion on fungal hyphae (86–90). Hyphal exudates contain compounds similar to those detected in plant root exudates, including amino acids, carboxylates, and carbohydrates (91), so a role for bacterial chemotaxis in association with the surface of hyphae would not be surprising.

Bacterial flagella are typically required for the movement of motile bacteria within a thin water film around the hyphae (79, 92–94). Chemotaxis toward fungal hyphal exudates was also proposed to promote the association of motile bacteria with hyphal surfaces for dispersion (91). However, non-motile bacteria that can adhere to fungal hyphae can also be dispersed through the fungal highways by adhering to the hyphal surfaces (91, 95–99). Some bacterial species can even switch from moving along the hyphal water film using flagella to becoming adherent to the mycelial surfaces via type IV pili or bacterial extracellular matrix components (79, 93, 95–98). Hyphal networks promote dispersion of diverse bacteria under conditions that otherwise limit bacterial swimming or swarming, such as under low water saturation conditions (92). In addition, hyphal surface hydrophobicity may modulate the propensity of hyphal highways to support bacterial movement by determining whether a continuous water film exists on the hyphal surfaces and the thickness of the water film (86). The growing tips of hyphae are more hydrophilic because the cell walls are thinner. As the hyphae age, the cell walls thicken, and so does the surface of the hyphae, which becomes more hydrophobic, which could reduce the ability to retain water on the hyphal surfaces (86). These temporal changes could also explain that bacteria may switch between motility and attachment to the hyphae and/or use multiple modes of adhesion to the fungal surfaces for dispersion.

Evidence also points to flagellated bacteria exploiting fungal highways for long-distance dispersion in the soil and, upon reaching the rhizosphere, switching to colonization of root surfaces. This is the case of the biocontrol bacterium *Rahnella aquatilis*, which produces gluconic acid in the rhizosphere of tomato plants and, in doing so, inhibits *Fusarium oxysporum*-mediated alkalinization, which causes plant disease (90). *R. aquatilis* is chemotactically attracted to *F. oxysporum* hyphae and moves up an alkaline pH gradient originating from the fungal hyphae and toward the hyphal surfaces. Using flagellar motility, *R. aquatilis* moves along the hyphae for long-distance transport toward tomato roots that attract *F. oxysporum* by chemotropism. Once in the vicinity of tomato roots, *R. aquatilis* moves toward the hyphal tips to colonize the root surfaces. Once in the rhizosphere, *R. aquatilis* produces gluconic acid and acidifies the rhizosphere to protect the plant from the fungal-mediated alkalinization, which the fungal pathogen uses as a general virulence factor. There is recent evidence that other biocontrol *Bacillus* spp. strains are attracted to *F. oxysporum* hyphae and that they could utilize similar fungal highways for dispersion and plant root surface colonization (100) or to promote predatory bacteria access to their prey (101). Similarly, *Sinorhizobium meliloti* can colonize legumes and nodulate its legume host, *Medicago truncatula*, using long-distance transport via the extraradical mycelium of the arbuscular mycorrhizae *Rhizophagus irregularis* (102). Several studies have also reported evidence of bacterial migration on the surface of fungal hyphae, suggesting it is a major mode of long-distance transportation of bacteria in the soil (80, 86, 102). However, direct evidence for the role of bacterial chemotaxis (e.g., comparing chemotactic wild-type vs non-chemotactic mutants and the identification of specific chemoreceptors involved) remains to be obtained. The predicted size of the belowground hyphal network suggests that dispersion of bacteria within the soil through hyphal highways is common. Dispersion of motile flagellated bacteria through fungal highways was proposed to explain the persistence of flagellar motility in soil environments that are not sufficiently water-saturated to support flagellar motility-mediated bacterial dispersion (92). However, much remains to be learned about the diversity of mechanisms by which bacteria detect and associate with fungal hyphae and the ecological impact that the movement along hyphal highways may have on soil microbial community dynamics.

## MOVEMENT EMERGING FROM INTERSPECIES BACTERIAL INTERACTIONS

Motility of bacteria has been mostly studied using monocultures under controlled laboratory conditions, in liquid or solid media. However, ongoing evidence in the literature points to movement as an emerging behavior resulting from interaction between bacteria of different species or genera. For example, under conditions of nutrient limitation and in the presence of a yeast strain competitor, *Streptomyces venezuelae* induces an exploratory growth that permits rapid movement across both biotic and abiotic surfaces (103, 104). Strikingly, the exploratory growth is associated with higher pH conditions that result from the production of the trimethylamine volatile compounds (VOC) by *S. venezuelae*. This VOC also acts as a long-distance signaling molecule to induce exploratory growth in physically distant *Streptomyces* (103, 105). The VOC trimethylamine itself increases the environmental pH and suppresses the growth of other microorganisms because the alkalinization of the medium reduces iron availability (103, 105). Similarly, the subinhibitory production of chloramphenicol by *S. venezuelae* co-cultured with *B. subtilis* promotes movement by sliding motility (106). Sliding in *B. subtilis* is a flagellum-independent motility on agar surfaces that depends on growth, as well as the production of surfactin and EPS that are thought to condition the surfaces and promote water retention to facilitate rapid surface growth (107). Similar patterns of *B. subtilis* mobilization were also observed in co-culture with other *Streptomyces* species (106). Other translation-inhibiting antibiotics, often produced by *Streptomyces* species, also induced sliding in *B. subtilis*, suggesting that translation stress induced a growth-dependent mobilization of the *B. subtilis* population of cells (106). Similarly, the presence of plant-associated *Pseudomonas* species capable of producing toxic cyclic lipopeptides induced surfactin production and sliding motility of *Bacillus velezensis* (108). Rapid movement across surfaces by sliding motility or exploratory growth could thus represent adaptive responses to rapidly move away from antimicrobial toxic compounds produced during interspecies competitive interactions. This adaptive response could be especially relevant in environmental conditions that do not support robust swimming or swarming, such as plant or soil aggregate surfaces or in multi-species biofilms. Sliding motility can also exist in the absence of competitive interactions. For example, sliding motility has been described in a monoculture of *B. subtilis* (109). *Sinorhizobium meliloti* (110), the plant pathogens *Erwinia amylovora* (111) and *Pseudomonas syringae* pv. tomato (112). In many models, EPS is thought to drive sliding movement by retaining moisture and thus modulating osmotic pressure. Sliding in monocultures has been previously reviewed (113).

The term “social spreading” is used to describe the co-migration of bacteria in close contact in a mixed colony across a hard agar surface under conditions where none of the individual species is motile (114). This form of movement has been observed between some strains of *Pedobacter* sp. and some strains of *P. fluorescens*, and thus appeared to emerge from interactions between strains of the *P. fluorescens* group and strains in the *Pedobacter* genus (115). Depending on the strains mixed together, co-migration emerged through diffusible signals, while for other strains, co-migration depended exclusively on close cell-to-cell contacts (115). Social spreading required *P. fluorescens* flagellum and flagellar function, suggesting that *Pedobacter* modifies the local environment where the two strains interact to allow *P. fluorescens* to move using flagellar motility under non-permissive (hard agar surface) conditions (116). Social spreading resulting from closely interacting strains was induced under nutrient-limited conditions and could also be enhanced with increased osmolarity (salts or monovalent cations added), suggesting a critical role for the environment, and thus microbial metabolism, on the emergence of social spreading (115). The observations are remarkable for several reasons. First, the variability in the emergent motility behavior between combinations of closely related strains of *Pedobacter* and closely related strains of the *P. fluorescens* group hints that the behavior is likely fine-tuned to environmental conditions and metabolic status of the interacting strains. Second, these observations highlight the general dearth of understanding of interspecies interactions relevant to the bacterial diversity in the soil environment and the potential myriads of strain combinations capable of producing emergent (motility) behaviors.

The emergent motility behaviors between interacting bacterial species described above all occur between bacterial strains and in the presence of additional stressors, such as low nutrients, increased osmolarity, or the presence of antimicrobial compounds. These stressors are prototypical of soil conditions, suggesting that the emergent motility behaviors such as those described above are common in soil and rhizosphere niches where multiple species interact.

## CONCLUSIONS AND OUTLOOK

As is true for many soil and rhizosphere processes, knowledge about the contribution of the various mechanisms described above to the dispersion of bacteria in the soil and rhizosphere remains limited. The few model systems that have been characterized offer a window into the many modes by which bacteria may be transported passively or actively within the soil and in the rhizosphere. Critical factors for movement include soil properties (pore structures, size, and connectivity), water availability, and microbial diversity. Soil pore microbiomes and their activity are major drivers of soil biogeochemistry, with plant roots influencing soil pore architectures (2). The ranges of speed for the different modes of movement suggest dispersal of bacteria in the soil occurs at multiple scales (Table 1), with the dispersed distance also depending on soil physicochemical characteristics, including pores connectivity and water hydraulic conditions (Fig. 1). Lack of quantitative information about the impact of bacteria dispersal processes on local soil pore microbiome community structures and function (8) limits our ability to accurately model soil biological processes. Emerging forms of motility that result from microbe-microbe interactions have also been recently described, and the few examples discussed here suggest that microbial secondary metabolites and soil conditions trigger movements in interspecies interactions that largely remain underexplored. The dispersion of bacteria through fungal highways appears to have the most potential to move bacteria over long distances in the soil (Fig. 1). While evidence indicates that arbuscular mycorrhizae specifically attract some motile bacteria to their hyphae through exudates (86), whether exudates from other fungi are able to specifically attract bacteria remains to be determined. The growing body of literature on chemotaxis in soil bacteria, including the identification of chemoreceptors-sensory specificity (117), could help address the hypotheses regarding the attraction of motile bacteria to fungal hyphae. While the biogeography of soil bacterial operational taxonomic units (7, 8) suggests limited dispersal, this taxonomic level doesn’t resolve dispersals of strains and subpopulations that could influence the structures and function of local bacterial communities. The range of speed of biological movements suggests potential for both local and long-distance active and passive modes of dispersal for microorganisms (Table 1). High-resolution mapping of bacteria in soil and in the rhizosphere, combined with real-time analytical methods to identify bacterial activity and function, would allow the quantitative evaluation of the contribution of various modes of dispersal and their impact on soil and microbial community structures and function. This knowledge is critical to better understand the evolution of soil bacterial communities, the impact of agricultural practices, and the effect of inoculation and soil amendments on soil productivity.
